# How to Succeed in Our Profession

**Published:** 1880-03

**Authors:** 


### HOW TO SUCCEED IN OUR PROFESSION.

To succeed in our profession, to attain the very highest position, winning " golden opinions," as well as *securing* "*golden ducats*," and "after life's fitful fever" to leave behind a name worthy of our deeds, should be an inspiration in every man's bosom. The desire for success, to stand ahead in the ranks, "to be a hero in the strife," has been in all ages the grand motive principle in the accomplishment of all worthy undertakings. Nothing of merit is obtainable without mental and physical labor ! The man who waits, Micawber like, " for something to turn up," will find that he is left by the wayside, if not trampled under and vanquished by others pushing and struggling along the highway to success. In this day of advancement, when every facility is offered, when the very means are, as it were, thrust upon us to make us successful, there is no just excuse for any one lagging or falling by the wayside.

Ours is a noble calling. We are in one sense humanitarians. To alleviate pain, to restore lost parts, is indeed a worthy mission. To accomplish these ends most pleasantly, most intelligently and most satisfactorily, should be our constant aim.

The first great element to success is a thorough education, to be sufficiently informed in leading sciences; but especially to be thoroughly posted on all branches of our profession, are marked requisites. One who accepts the responsibilities of a profession without such qualifications is unworthy the exalted relationship he seeks. The effort to lay first a broad basis for a professional superstructure may cost self-denial, self-sacrifice, toil and struggles; but the goodness of learning rightly rewards all who pay their devotions at her altars.

Professional prominence and business success are becoming every year more dependent upon a thorough collegiate education. If we consider our life-work in a mere business, financial sense, statistics show that educated mind has greatly the advantage, and that the time and money spent in colleges are both a saving of time and investment richly productive. Business men now bear testimony to the fact that education is of great service to merchants, publishers, mechanics, agriculturists, etc. In the profession there is every need for cultured mind. Only such rise to anything like commanding usefulness.

Education is much more general now among the people, and they will demand more of all who strive to lead or instruct them. We are told that all professions and vocations are crowded; but there never was such a demand and such opportunities for thoroughly trained intellect and ripe scholarship. Hence many doors of usefulness and of pre-eminence open to every one giving any evidence of superior qualifications. Our profession is peer to any in offering facilities for acquiring a thorough knowledge of all her branches. A number of colleges are established here and there, presided over by the brightest lights, where all who aspire can go and be thoroughly cultured and skillfully trained. We have many journals, edited and contributed to by men of ability, and through them much valuable information can be gleaned.

Taking it for granted that this first and essential step has been taken on the road to success, there is more yet to do. Education, without the full exercise of it, is a stand-still. The intelligent dentist will read his journals, note the improvements, experiment, and be thoroughly up with the times. To succeed, he must be honest, industrious and persevering. He must never lag in his efforts to improve and give his patrons the very best skill he can possibly acquire. The day for the slow, plodding dentist is passing. This is an age of wonderful enlightenment and improvement, and the man who contents himself with the practice of a decade, aye, a year passed, will surely be left behind by his more enterprising compeers. People demand improvements, and will patronize the skillful dentist who furnishes them. Where is the man, woman or child who will sit in a dental chair for hours having a tooth operated upon, suffering in "wear and tear" of nerve force to the exhaustion of strength and patience, when by new methods and improvements the same work can be accomplished in one-tenth the time, with far less pain and decidedly more comfort? The means are accessible to every dentist, and he is acting against his own interests who does not secure them. Every dentist should have an S. S. White or Johnston Brothers' improved engine. They are wonderful aids in doing first-class work. They save time, expedite work, and make the operation less painful; in short, after being once used, no dentist would surrender them for their weight in gold. Mr. White and Johnston Brothers have spent thousands of dollars in perfecting their wonderful machines, and are now offering them to the profession at a trifle compared to the value derived from them. To facilitate work and make operations less wearisome and wearing, every dentist should have a modern dental chair. Men of genius have been improving on them each year, until now they are brought to almost a state of perfection. For beauty of design, for perfection in giving positions and simplicity of movements, no chair can excel the Wilkerson chair, invented by Dr. Wilkerson, of the Baltimore Dental College, and manufactured by the Johnston Brothers, New York. * * *

If we would strive to do only first-class work—securing favor and patronage by perfection in operations rather than by cheap competition—how much more like professional men we would be, and how much more respect we would win from our patrons! Our profession should not be lowered by making it a bartering business, but rather elevated by showing to the people that we respect our calling and professional attainments. It is the curse of the day, this selling our wear and tear of mental and physical forces for a mere pittance. Let us, then, strive to perfect ourselves in the knowledge of all branches of our profession. Let us avail ourselves of all improvements of the day, and present ourselves to the people as professional men worthy of their patronage and just compensation. *If we do this*, we must and will succeed—be an ornament to our profession—

"And, departing, leave behind us
Footprints on the sands of time."

—*Dental Luminary*, *Macon*, *Ga.*

